# A multiscale imaging approach to studying the harpoon-like invasion organelle from microsporidian pathogens

**DOI:** 10.1063/4.0001000

**Published:** 2025-10-27

**Authors:** Gira Bhabha1, Nicolas Coudray, Mahrukh Usmani, Margot Riggi, Janet Iwasa, Damian C Ekiert, Rishwanth Raghu, Ellen Zhong, Harshita Ramchandani, Daija Bobe, Mykhailo Kopylov

**Affiliations:** 1 Johns Hopkins University, Baltimore, MD, USA; 2 Max Planck Institute of Biochemistry, Munich-Martinsried, Bavaria, Germany; 3 University of Utah, Salt Lake City, UT, USA; 4 Princeton University, Princeton, New Jersey, USA; 5 Princeton University, Princeton, NJ, USA; 6 University of Michigan, Ann Arbor, MI, USA; 7 New York Structural Biology Center, New York, NY, USA

## Abstract

Microsporidia are tiny, single-celled parasites similar to fungi that infect a wide range of animal species, from worms and honey bees to humans. In humans, these opportunistic pathogens can cause life-threatening infections in immunocompromised individuals. To initiate an infection, microsporidia harness a specialized harpoon-like invasion apparatus called the polar tube (PT) to gain entry into host cells. The PT is tightly coiled within the transmissible extracellular spore, and is about 20 times the length of the spore. Once triggered, the PT is rapidly ejected, within milliseconds, and is thought to penetrate the host cell, acting as a conduit for the transfer of infectious cargo into the host, to initiate infection. Once inside host cells, microsporidia create a niche which is permissive to their development. We combine optical microscopy, Volume electron microscopy and structural cell biology to decipher the 3-dimensional organization, dynamics, and mechanism of the polar tube, parasite development, and host- parasite interactions.

